# Rates of Anti-Tuberculosis Drug Resistance in Kampala-Uganda Are Low and Not Associated with HIV Infection

**DOI:** 10.1371/journal.pone.0016130

**Published:** 2011-01-10

**Authors:** Deus Lukoye, Frank G. J. Cobelens, Nicholas Ezati, Samuel Kirimunda, Francis E. Adatu, Joseph K. Lule, Fred Nuwaha, Moses L. Joloba

**Affiliations:** 1 Public Health Department, Kampala City Council, Kampala, Uganda; 2 Center for Poverty-Related Communicable Diseases, Amsterdam Institute of Global Health and Development, Academic Medical Centre, Amsterdam, The Netherlands; 3 National Tuberculosis and Leprosy Programme, Kampala, Uganda; 4 Department of Medical Microbiology, School of Biomedical Sciences, Makerere University College of Health Sciences, Kampala, Uganda; 5 Makerere University School of Public Health, Makerere University College of Health Sciences, Kampala, Uganda; University of Otago, New Zealand

## Abstract

**Background:**

Drug resistance among tuberculosis patients in sub-Saharan Africa is increasing, possibly due to association with HIV infection. We studied drug resistance and HIV infection in a representative sample of 533 smear-positive tuberculosis patients diagnosed in Kampala, Uganda.

**Methods/Principal Findings:**

Among 473 new patients, multidrug resistance was found in 5 (1.1%, 95% CI 0.3–2.5) and resistance to any drug in 57 (12.1%, 9.3–15.3). Among 60 previously treated patients this was 7 (11.7%, 4.8–22.6) and 17 (28.3%; 17.5–41.4), respectively. Of 517 patients with HIV results, 165 (31.9%, 27.9–36.1) tested positive. Neither multidrug (adjusted odds ratio (OR^adj^) 0.7; 95% CI 0.19–2.6) nor any resistance (OR^adj^ 0.7; 0.43–1.3) was associated with HIV status. Primary resistance to any drug was more common among patients who had worked in health care (OR^adj^ 3.5; 1.0–12.0).

**Conclusion/Significance:**

Anti-tuberculosis drug resistance rates in Kampala are low and not associated with HIV infection, but may be associated with exposure during health care.

## Introduction

An estimated 9.3 million incident tuberculosis (TB) cases and 1.4 million deaths occurred in 2007, making TB a major cause of morbidity and mortality in the world. Of major concern to TB control is the resistance to first-line anti-tuberculosis drugs. In particular with multi-drug resistance (MDR), i.e resistance to isoniazid and rifampicin, cure rates for first-line anti-TB drug treatment are significantly reduced [1], [2].

WHO estimates that 490,000 MDR-TB cases emerge every year, representing 5.3% of all TB cases globally, resulting in 110,000 deaths [3]. Data on anti-TB drug resistance for sub-Saharan Africa are often limited to hospital settings and representative data based on quality-assured susceptibility testing are scarce [2]. These data tend to show low MDR rates, but concerns have been recently fuelled by an epidemic in Southern Africa of MDR-TB, compounded by resistance to major second-line drugs (extensively drug-resistant/XDR-TB), emerging against a background of high HIV prevalence and expanding antiretroviral treatment [2], [4].

HIV has a strong impact on TB incidence rates and HIV infection has been associated with MDR-TB through outbreaks in institutional settings [5]. Most studies however date from before the large-scale roll-out of antiretroviral treatment (ART). Since ART strongly reduces mortality in co-infected patients, it may have a paradoxical effect of increased transmission of MDR-TB through prolonged survival of infectious MDR-TB patients [6]. Results of the Uganda HIV sero-behavioral survey (2005) showed an HIV prevalence of 8.5% for Kampala district and ART has been widely rolled out in the district since 2004 (Kampala City Council, unpublished data).

In the first anti-TB drug resistance survey in Uganda (1996–1997), which used a similar sampling scheme as ours but covered another area of the country, the prevalence of resistance to any drugs was 19.8% and of MDR-TB 0.5% [7]. In a hospital-based study conducted two years later in Kampala, MDR prevalence was the same (0.5%), but resistance to rifampicin was 1.4%. More recently a hospital based study in Kampala found 12.7% MDR among previously treated patients [8]. Therefore data on anti-TB drug resistance in Kampala was either outdated or hospital based and no risk factors for anti-TB drug resistance were known.

To be able to estimate the current prevalence of anti-TB drug resistance and risk factors associated with it in Kampala district, we carried out a cross-sectional survey among new and previously treated TB patients. We in particular wanted to establish whether drug resistance prevalence among new cases had increased and/or become associated with HIV status over the past 10 years during which anti-retroviral treatment has been rolled out, increasing from 44,000 in 2004 to 193,000 in June 2009(Uganda AIDS Control Programme un published data).

## Methods

Ethical approval was obtained from the Research and Ethics Committees of Makerere University College of Health Sciences, Kampala. Written informed consent was obtained from all the patients who participated in the study.

### Study design

We conducted this survey between 18 August and 19 December 2008 in all health care facilities in Kampala that reported TB cases to the National TB/Leprosy Programme (NTLP). This period was determined by the sample size of 483 new patients, based on the requirement to measure in this group an MDR prevalence of 1.4% with an upper boundary of the 95% confidence interval of 3.0%. Since all heath care facilities were included in the sample, we assumed a design effect of 1. For logistical reasons we grouped the facilities into three, based on the number of sputum smear-positive patients expected in each group. Patient enrollment followed a rotational fashion from one group of health facilities to another so that each group participated for the same period in the study, thus keeping a self weighted sample All sputum smear-positive patients who were registered for treatment during the enrollment period aged 18 years or above and consented to participate were enrolled.

### Data collection

Each participant who consented to participate was requested to provide two sputum samples (an early morning and spot) and a blood sample for HIV testing. HIV testing was done within 24 hours of collection at a quality-assured laboratory, independently of the routine HIV counseling and testing procedures.

Information about demographic characteristics, HIV status prior to enrollment, use of anti-retroviral treatment and history of TB treatment was collected through a structured interview. We also obtained data about risk factors for anti-TB drug resistance and HIV, including prison and health care exposure, injection drug use and involvement in commercial sexual activities.

We defined a patient as previously treated if this patient had a history of having taken first-line anti-tuberculosis drugs for one month or more and as new if otherwise. Patient treatment history was ascertained at the health facility using a standardized questionnaire recommended by WHO [9].

We carried out re-interviews on 110 (20%) participants randomly selected from the enrolled patients within 6 weeks of the original interview to check for the quality of ascertainment of treatment history. Re-interviews were conducted by staff who were independent of the clinic where data was collected and blinded to the original interview result; none showed any discrepancy in treatment history classification to the original interviews.

### Laboratory methods

#### Sputum culture

For each included patient the sputum specimen with the highest bacillary count was decontaminated and inoculated onto two slopes: one glycerol and the one pyruvate Löwenstein-Jensen (L-J) medium incubated at 35–37°C and examined weekly for growth up to 8 weeks. Cultures showing no growth at 8 weeks were reported as negative. *M. tuberculosis* identification was based on presumptive appearance of colonies on culture and later confirmed by IS6110 based PCR. In addition, when performing drug susceptibility testing, paraminobenzoic Acid (PNB) 500 µg/ml was used to differentiate non-tuberculous mycobacteria from *M. tuberculosis*.

#### Drug susceptibility testing (DST)

All *M. tuberculosis* isolates were tested at the National TB Reference Laboratory for resistance to isoniazid, rifampicin, ethambutol and streptomycin by the L-J proportion method using as concentrations 0.2 µg/ml for isoniazid, 4 µg/ml for rifampicin, 40 µg/ml for ethambutol, and 2.0 µg/ml for streptomycin. MDR was defined as resistance of an isolate to at least isoniazid and rifampicin. Second line DST was done on all MDR isolates using LJ proportional method using critical concentrations 2.0 µg/ml for ofloxacin and 30.0 µg/ml for kanamycin [10].

Drug susceptibility proficiency testing was performed at the Supranational Reference Laboratory (SRL) in Borstel (Germany) on all the identified MDR isolates, on 15 isolates randomly selected from the remaining isolates of previously treated patients and on all rifampicin-monoresistant isolates.

#### HIV Testing

HIV antibody testing was done in parallel using Abbot Determine (Abbott Laboratories Abbott Park IL, USA) and double-well run Vironostika HIV Uni-form II Ag/Ab (BioMerieux Boxtel, Netherlands). The Generic Biorad HIV-1/HIV-2 plus O-ELISA kit (Biorad Laboratories, Redmond WA, USA) was used as the tie-breaker. All tests were performed in accordance with the manufacturers' instructions.

### Data management

Data were double entered into Epidata 3.1 (www.epidta.dk). Discrepancies were checked against the raw data. Analyses were done in STATA v10 (Stata Corp. College Station TX, USA). For comparison of categorical variables we used the X^2^ test or the 2-sided Fishers' exact test as appropriate. Uni- and multivariable analyses were done by logistic regression. Contribution of the variables to the model was tested using the likelihood ratio X^2^ test. All significance testing was done at 5% confidence level.

## Results

During the rotation periods, 633 sputum smear-positive TB patients were registered with the NTLP of whom 557 (87.9%) were enrolled after submission of 2 sputum samples. Most of the non enrollments were due to declining participation and late start of enrollment due to administrative problems in some of the participating units. These did not significantly differ with regard to demographic characteristics like age, sex or history of previous TB treatment. None of the cultures grew non-tuberculous mycobacteria.

Table 1 shows characteristics of the patients and proportions with complete data.

**Table 1:** Characteristics of new and previously treated TB patients diagnosed in Kampala; August-December 2008.

PATIENT Characteristic		Enrolled n = 557 (%)**
Sex	male	327 (58.7)
	female	230 (41.3)
Age	18–24	183 (32.8)
	25–34	216 (38.8)
	35–44	108 (19.4)
	45–54	37 (6.6)
	> = 55	13 (2.3)
Highest level of Education	Non	36/557 (6.5)
	Primary	228/557 (40.9)
	Secondary	228/557 (40.9)
	Higher learning	37/557 (6.6)
	Unknown	4
Marital Status	Single	216/557 (38.8)
	Married	210/557 (37.7)
	Separated	82/557 (14.7)
	Widowed	23/557 (4.1)
	Cohabiting	21/557 (3.8)
	None of the above	5 (0.9)
Employment status	Public Servant	34 (6.1)
	Self employed	430 (77.2)
	Peasant	41 (7.3)
	Student	52 (9.3)
Residence	Kampala	373 (67.0)
	Outside Kampala	184 (33.0)
†HIV infection previously diagnosed positive	Yes	96(16.6)
ART* use at enrolment	Yes	34 (5.8)
Previous history of TB treatment	Yes	62 (11.1)
	No	495 (88.9)

**Column percentages.

† HIV =  Human Immunodeficiency Virus.

*ART =  Anti-Retroviral Therapy.

Of the study participants 327 (58.7%) were male, the modal age group was 25–34 years (216 participants, 38.8%), 50 (8.9%) were 45 years and above. There were 495 (88.9%) new and 62 (11.1%) previously treated patients. Three hundred and seventeen (59.5%) participants had been tested for HIV in the past; 91 (28.7%) were known to be HIV-positive. Of these, 34 (37.4%) were on anti-retroviral treatment; only one participant had used INH prophylaxis before (table 1).

Out of the 557 smear-positive specimens received at NTRL, those from 12 (2.2%) participants were contaminated and 12 (2.2%) were culture-negative. Therefore DST results were available for 533 (95.6%) patients of whom 473 (88.7%) were new and 60 (11.3%) previously treated (table 2). Of the latter the outcome of previous treatment had been cure for 20, treatment completion with no smear results for 16, failure for 7 and default for 17.

**Table 2:** Anti-TB drug resistance among new and previously treated cases in Kampala; August-December 2008.

	New cases(473)		Previously treated TB cases (60)		All cases	
Pattern of resistance	Number (%)	95% CI	Number (%)	95% CI	Number (%)	95% CI
Total Patients	473 (88.7)		60 (11.3)		533	
Susceptible to all	416 (87.9)	84.7–90.6	43(71.6)	58.5–82.5	459 (86.1)	82.8–89
Any resistance*	57 (12.2)	9.2–15.3	17 (28.3)	17.4–41.4	74(13.9)	11.0–17.1
Any resistance to;						
RMP	7 (1.5)	0.5–3.0	8 (13.3)	5.9–24	15 (2.8)	1.5–4.5
INH	27 (5.7)	3.8–8.2	12 (20)	10.7–32.0	39 (7.3)	5.2–9.8
EMB	3 (0.64)	0.13–1.8	6 (10)	3.7–20.5	9 (1.7)	0.7–3.1
SM	41 (8.7)	6.3–11.5	9 (15)	7–26.5	50 (9.4)	7–12.2
H+R Resistance (MDR**)						
INH+RMP	4 (0.85)	0.23–2.1	2 (3.3)	0.4–11.5	6 (1.1)	0.4–2.4
INH+RMP+EMB	1(0.21)	0.54–11.7	1(1.7)	0.04–8.9	2(0.4)	0.04–13.5
INH+RMP+SM	0(0)	-	2(3.3)	0.4–11.5	2 (0.4)	0.04–13.5
INH+RMP+EMB+SM			2 (3.3)	0.4–11.5	2 (0.4)	0.04–13.5
INH+ other resistance						
INH+EMB	1 (0.21)	0.54–11.7	4 (6.7)	1.8–16	5 (0.94)	0.3–2.1
INH+SM	12 (2.5)	1.3–4.3	4 (6.7)	1.8–16	16 (3)	1.7–4.8
INH+EMB+SM	0(0)	-	2 (3.3)	0.4–11.5	2 (0.4)	0.04–13.5
RMP+ other resistance						
RMP+EMB	1 (0.21)	0.54–1.7	4 (6.7)	1.8–16.0	5 (0.94)	0.3–2.1
RMP+SM	2 (0.42)	0.05–1.5	5 (8.3)	2.7–18	7 (1.3)	0.5–2.6
RMP+EMB+SM	0(0)	-	1(1.7)	0.04–8.7	1(0.2)	0.005–10
Monoresistance‡						
RMP	0(0)	-	0(0)	-	0(0)	-
INH	10 (2.12)	1.0–3.8	4 (6.7)	1.8–16	14 (2.6)	1.4–3.3
EMB	1 (0.21)	0.054–1.7	0(0)	0(0)	1 (0.19)	0.0004–0.010
SM	26 (12.9)	10–16	3(5)	1–13	29 (5.4)	3.6–7.7
Other resistance†						
EMB+SM	1 (0.21)	0.054–1.7	4 (6.7)	1.8–16	5 (0.94)	0.3–2.1

*Any resistance: resistance to the drug with or without resistance to other drugs.

RMP = rifampicin, INH = Isoniazid, SM = streptomycin, EMB =  ethambutol.

CI  =  Confidence Interval.

**MDR: Multidrug resistance, i.e. resistance to at least INH and RMP.

‡ Monoresistance: resistance to only one anti-tuberculosis drug.

† Other resistance: Resistance to a combination of other drugs not including INH or RMP.

Among the new cases, MDR (percentage, 95% confidence interval (CI)) was found in 5 (1.1; 0.3–2.5) and any drug resistance in 57 (12.1, 9.3–15.3). Resistance among new cases was most frequent to streptomycin (8.7%) and isoniazid (5.7%).

Among the 60 previously treated cases, 7 (11.7, 4.8–22.6) had MDR and 17 (28.3, 17.4–41.4) had any resistance. Of the 7 MDR cases among this category, 3 (42.8%) were resistant to all four drugs, 2 (28.6%) were additionally resistant to streptomycin and 2 (28.6%) were resistant to isoniazid and rifampicin only.

Among new and previously treated cases combined, 13.9% (95% CI 11.0–17.1) had any resistance, 2.3% (95% CI 1.2–3.9) had MDR, 9.3% (95% CI 7.0–12.2) had mono-resistance and 3.4% (95% CI 2.0–5.3) had poly-resistance. Specific resistance patterns are shown in table 2.

Of the 30 samples submitted for external quality assessment, susceptibility results were concordant for 28 (93.3%) of the isolates. All resistant isolates were correctly detected, 2 isolates which were initially monoresistant to rifampicin turned out pansensitive at retesting.

Of the 517 patients with HIV results 165 (31.9%, 95% CI 27.9–36.1) tested positive. No association was established among new or previously treated patients between any drug resistance and HIV status, neither before (table 3) nor after adjustment for potential confounders (table 4). Nor did we find any association between MDR and HIV status, although numbers were too small to allow meaningful multivariable analysis. We did not find significant associations between any drug resistance or MDR and any of the other demographic variables or potential risk factors for TB drug resistance or HIV infection, including anti-retroviral treatment among the HIV infected and history of hospitalization. We did however find a significantly increased risk of any resistance among new patients who had a history of having worked in health care (adjusted odds ratio 3.5 95% CI 1.0–12.2; p = 0.045). All 14 patients with a history of health care work had been tested for HIV. Of the 4 who had any drug resistance, 2 (50%) were HIV-infected, compared to 2 of 10 who had not (2-sided Fisher's exact p-value  = 0.520), while there was no increased prevalence of HIV infection among patients with a history of health care work (4 of 10, 28.6%) compared to those without (161 of 503, 32.0%, p>0.999). Two had monoresistance to streptomycin, one monoresistance to isoniazid, and one combined resistance to streptomycin and isoniazid.

**Table 3:** Univariable analysis for risk factors for any anti-TB drug resistance in Kampala.

Characteristic	New patients	Previously treated patients	
	n/N%	OR; 95% CI	P	n/N (%)	OR; 95% CI	P
Sex						
Males	29/278 (10.4)	1	0.200	14/37 (37.8)	1	0.045
Females	28/195 (14.4)	1.44 (0.83–2.51)		3/23 (13.0)	0.25 (0.06–0.98)	
Age (years)						
15–24	20/166 (12.1)	1	0.763	3/11 (27.3)	1	0.620
25–34	21/185 (11.4)	0.93 (0.49–1.79)		5/22 (22.7)	0.78 (0.15–4.12)	
35–44	12/84 (14.3)	1.22 (0.56–2.63)		6/18 (33.3)	1.33 (0.26–6.94)	
45–54	4/27 (14.8)	1.27 (0.40–4.05)		2/8(25)	0.89 (0.11–7.11)	
≥55	0/11	-		1/1		
HIV infection status						
Negative	40/308 (13.0)	1	0.444	14/44(31.8)	1	0.518
Positive	15/149 (10.1	0.75 (0.40–1.41)		3/16(18.8)	049 (0.12–2.02)	
Worked in Health care						
No	53/461 (11.5)	1	0.045	17/58 (29.3)	1	
Yes	4/12 (33.3)	3.85 (1.12–13.2)		0/2	-	
Admitted to hospital						
No	50/422 (11.9)	1	0.652	12/42(28.6)	1	1.000
Yes	7/51 (13.7)	1.18 (0.51–2.77		5/18(27.8)	0.96 (0.28–3.29)	
History of imprisonment						
No	56/435 (12.9)	1	0.069	14/53 (26.4)	1	0.393
Yes	1/38 (2.6)	0.18 (0.02–1.36)		3/7 (42.9)	2.09 (0.41–10.5)	

*See table 2 above for definitions.

**Table 4:** Multivariate analysis for risk factors associated with any resistance to anti-TB drugs in Kampala.

NEW PATIENTS				
Any resistance	resistant	Number (%)	OR (95% CI)	P-Value
HIV positive	Yes	15/149 (10.1)	0.7 (0.4–1.3)	0.46
	No	40/308 (13)		
Worked in a health care setting	Yes	4/12 (33.3)	3.5(1. –12)	0.045
	No	53/461 (11.5)		

Previously treated patients				
Any resistance	No	3/16	0.6 (0.12–2.9)	0.51
	Yes	14/44		

Other variables used for adjusting included age, sex, patient category, and marital status.

## Discussion

This study shows low prevalence of anti-TB drug resistance in Kampala district when new and previously treated patients are combined. Among previously treated patients, this is relatively high as established in other studies in the region. Seven of the twelve (58.3%) MDR cases were previously treated, yet this category contributed only 11.3% of the study participants. Although the MDR prevalence among new patients in this study was similar to that in a number of other studies carried out in the African region (1.4% in Burundi, 1.2% in Tanzania), it was lower than in Gambia (2.6%), Mozambique (3.4%) and Rwanda (3.9%) [11], [12]. This suggests that transmission of MDR-TB in Kampala is a limited problem. We found no association between any resistance or MDR and HIV infection. Although the number of MDR cases was small and thus limited the precision of our estimate, a clinically or epidemiologically relevant effect of HIV on the acquisition and/or transmission of MDR-TB would have resulted in sizable MDR prevalence among HIV-infected patients, which we did not observe.

The overall low MDR prevalence could result from community-based TB care with fewer chances of MDR-TB and HIV infected patients coming into close contact when seeking care in health facilities. In settings with high HIV prevalence, MDR outbreaks have been reported, generally resulting in increased anti-TB drug resistance prevalence among HIV infected patients [13]. Lack of association between drug resistance and HIV infection shows that opportunities for (nosocomial) transmission of drug-resistant TB may indeed be limited. In addition, supply of fixed dose combinations free of charge by the NTLP may contribute to patient adherence and prevent monotherapy during treatment. Finally the Uganda NTLP uses for adult new TB patients, who contribute about 80% of all the adult TB cases in Kampala, the eight-month standard regimen in which rifampicin is only given in the intensive phase. This regimen is likely to result in higher relapse rates than the six-month regimen where rifampicin is used throughout. However, it limits chances of improper use of rifampicin to the first two months, potentially reducing the risk of acquisition and hence transmission of MDR-TB in settings where observation of drug intake is not consistently applied throughout treatment.

Lack of association with HIV infection suggests that the recently initiated large-scale introduction of ART in Kampala has not resulted in paradoxical increases in rates of MDR-TB at the population level. It may be that such effects will not occur, or that they will only occur after prolonged large-scale use of ART, for example, because it may require several TB transmission cycles before an effect on MDR-TB transmission becomes apparent. Therefore, continued combined TB-drug resistance and HIV surveillance remains a priority in settings where TB and HIV coexist.

Worth noting in our findings is the relatively high prevalence of any resistance to streptomycin (15%) among previously treated patients, because streptomycin is used during the first 2 months in the standard retreatment regimen. Although NTLP is implementing routine HAIN based rapid DST for previously treated patients, the results are yet to be used in guiding treatment decisions.

Although HIV was not associated with anti-TB drug resistance, 32% of the study participants (and 26.7% of the previously TB treated participants) were HIV co-infected. However, TB/HIV co-infection among the patients notified to the NTLP is above 50% of those who are tested (NTLP, unpublished data). In the national referral hospital study mentioned earlier, the TB/HIV co-infection prevalence among previously treated patients was 49% [8], substantially higher than what we found. This may be due to a selection bias in the national surveillance data since not all notified TB patients are tested for HIV. In addition, TB patients diagnosed at the referral hospital may reflect a relatively ill selection, and therefore may have an increased probability of HIV infection. Finally, the wide roll-out of ART in Kampala to the level of primary care facilities may increase the average CD4 levels among the HIV infected, reducing the risk of progression from latent TB infection to TB disease. Further studies are required to explore whether HIV prevalence among TB patients has indeed declined in this setting and others where ART is provided at a large scale.

The association between transmitted anti-TB drug resistance and a history of health care work calls for confirmation in other studies since the numbers in this study were small: 4 patients with history of health care work showed drug resistance (to isoniazid and/to streptomycin) is cause for concern and calls for further exploration. It may reflect prolonged infectiousness of drug resistant cases due to delayed treatment response. This has been shown for resistance to isoniazid when current first line regimens are used, and may well occur in patients with streptomycin resistance when treated with the standard retreatment regimen [14]. As a result, health care workers could be more exposed to drug-resistant than to drug-susceptible TB. E.g when working on a TB ward or in another facility where TB patients on treatment are encountered. HIV infection among these patients may also have increased their risk of (re) infection and subsequent TB disease, however we found no difference in HIV prevalence between patients who did and patients who did not have history of health care work, either with or without drug resistance. We did not find HIV infection to be associated with having drug resistant TB among these health care workers.

### Limitations

This study had some limitations. The numbers of resistant cases, in particular MDR, were small, limiting the power to detect risk factors for drug resistance as statistically significant, including associations with HIV infection. However, if anything, we observed decreased risks of resistance among HIV-infected patients making it unlikely that we missed positive associations of substantial magnitude. While our sampling design in theory would result in consecutive sampling of all the eligible patient population, disrupted supply of anti-TB drugs during the survey, may have affected the sampling since some sites did not enroll participants into the study for the entire period. This could have led to selection bias if this problem was more frequent in clinics with relatively high resistance prevalence. For the same reason referral bias could not be ruled out, since larger health units were prioritized when anti-TB drug supplies were inadequate to cover all the TB treatment facilities. This probably did not significantly affect our results since health care facilities without drug supplies referred patients to other health units, and all TB care centers in Kampala were included in our study.

Anti-TB drug resistance prevalence in Kampala are low, but the occurrence of primary MDR indicating transmission of resistant *M. tuberculosis* strains is a threat to TB control in the district. We therefore recommend that directly observed therapy for diagnosed TB cases with drug-sensitive disease be strengthened to prevent acquired drug resistance and a country-wide drug resistance survey be carried out to establish the national burden. Establishment of Programmatic Management of Drug-resistant TB in the country is urgently required. The finding of health care work as a risk factor for drug-resistant TB as well as the continuing risk of outbreaks of (M)DR-TB among the HIV-infected call for strengthening TB infection control in health care settings to prevent nosocomial transmission. Routine DST of previously treated patients should be strengthened in order to identify MDR cases before treatment is started, since these have over 10% probability of having MDR-TB. The introduction of rapid resistance testing methods should be supported and treatment policies adjusted so that treatment of previously treated TB cases can be better guided by their resistance patterns.

### Conclusion and recommendations

Prevalence of anti-TB drug resistance in Kampala is low and not associated with HIV infection. Nonetheless continued and expanded surveillance of anti-TB drug resistance should be a priority. The association of drug resistance among new patients with a history of work in health care suggests nosocomial transmission and warrants further investigation.

**Figure 1:**
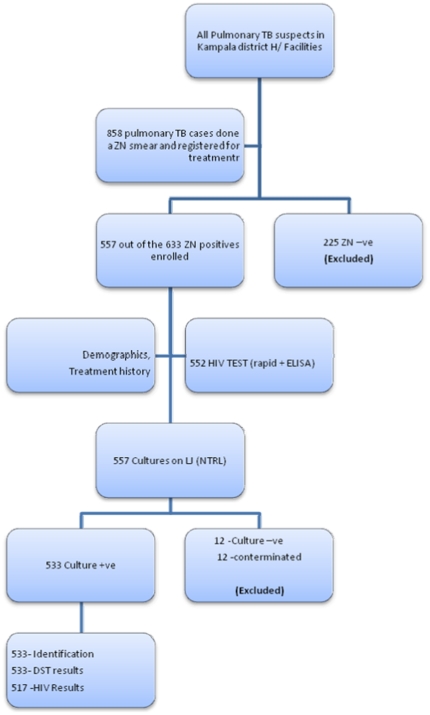
Patient flow chart during the study. DST, Drug Susceptibility testing; ZN –ve, Ziehl Neelsen negative; LJ, Lowenstein Jensen; Culture +ve, culture positive; Culture –ve, culture negative.
